# Prenylated Chalcone 2 Acts as an Antimitotic Agent and Enhances the Chemosensitivity of Tumor Cells to Paclitaxel

**DOI:** 10.3390/molecules21080982

**Published:** 2016-07-29

**Authors:** Joana Fonseca, Sandra Marques, Patrícia M. A. Silva, Pedro Brandão, Honorina Cidade, Madalena M. Pinto, Hassan Bousbaa

**Affiliations:** 1 CESPU, Instituto de Investigação e Formação Avançada em Ciências e Tecnologias da Saúde, IINFACTS, 4585-116 Gandra PRD, Portugal; joanafonsecabioq@hotmail.com (J.F.); marques.sandra075@gmail.com (S.M.); patricia_masilva@hotmail.com (P.M.A.S.); 2 Center for Biomedical Research, CBMR, University of Algarve, 8005-139 Faro, Portugal; 3 Departamento Ciências Biomédicas e Medicina, University of Algarve, 8005-139 Faro, Portugal; 4 Laboratório de Química Orgânica e Farmacêutica, Departamento de Ciências Químicas, Faculdade de Farmácia, Universidade do Porto, 4050-313 Porto, Portugal; pedrocgbrandao@gmail.com (P.B.); madalena@ff.up.pt (M.M.P.); 5 Centro Interdisciplinar de Investigação Marinha e Ambiental (CIIMAR/CIMAR), Universidade do Porto, 4050-123 Porto, Portugal

**Keywords:** prenylchalcone, antimitotic, mitotic spindle damage, apoptosis, paclitaxel chemosensitivity, drug resistance, cancer chemotherapy

## Abstract

We previously reported that prenylated chalcone 2 (PC2), the *O*-prenyl derivative (**2**) of 2′-hydroxy-3,4,4′,5,6′-pentamethoxychalcone (**1**), induced cytotoxicity of tumor cells via disruption of p53-MDM2 interaction. However, the cellular changes through which PC2 exerts its cytotoxic activity and its antitumor potential, remain to be addressed. In the present work, we aimed to (i) characterize the effect of PC2 on mitotic progression and the underlying mechanism; and to (ii) explore this information to evaluate its ability to sensitize tumor cells to paclitaxel in a combination regimen. PC2 was able to arrest breast adenocarcinoma MCF-7 and non-small cell lung cancer NCI-H460 cells in mitosis. All mitosis-arrested cells showed collapsed mitotic spindles with randomly distributed chromosomes, and activated spindle assembly checkpoint. Live-cell imaging revealed that the compound induced a prolonged delay (up to 14 h) in mitosis, culminating in massive cell death by blebbing. Importantly, PC2 in combination with paclitaxel enhanced the effect on cell growth inhibition as determined by cell viability and proliferation assays. Our findings demonstrate that the cytotoxicity induced by PC2 is mediated through antimitotic activity as a result of mitotic spindle damage. The enhancement effects of PC2 on chemosensitivity of cancer cells to paclitaxel encourage further validation of the clinical potential of this combination.

## 1. Introduction

Microtubules are hollow cylindrical polymers of the two globular proteins, α- and β-tubulin, and play a wide variety of essential dynamic and structural roles in eukaryotic cells [1]. For instance, they are involved in vesicle and organelle transport, cell motility and polarity, and also in cell division [1]. During mitosis, microtubules form the dynamic mitotic spindle are in charge of separating chromosomes between the two daughter cells. Because of this role in mitotic spindle structure and dynamics, disruption of microtubules induces chronic mitotic arrest and eventually cell death by apoptosis, making them a preferential target for cancer therapy [2]. Indeed, many of the known antimitotic compounds exert their cytotoxic activity by suppressing microtubule dynamics. These so-called Microtubule-Targeting Agents (MTAs) remain amongst the most effective chemotherapeutics used in the clinic, and include the widely used taxanes and Vinca alkaloids [3]. Taxanes, such as paclitaxel or docetaxel, enhance tubulin polymerization and act as microtubule stabilizing agents. Vinca alkaloids, such as vinblastine or vincristine, inhibit microtubule polymerization and act as microtubule destabilizers.

Despite the use of taxanes and Vinca alkaloids in managing many malignancies, their effectiveness has been limited by hematopoietic and neurologic toxicities due to the role of microtubules in non-tumor cells [3]. In addition, they face the problem of drug resistance due to upregulation of transmembrane efflux pumps that reduces their intracellular concentrations and thus their efficacy, and tubulin mutations that affect drug binding-site or induce aberrant expression of tubulin isotypes [4,5]. Nonetheless, given the overall therapeutic success of MTAs, research groups and pharmaceutical companies continue to invest in the development of new microtubule-targeting compounds that can overcome the disadvantages associated with the MTAs currently in use [6].

**Figure 1:**
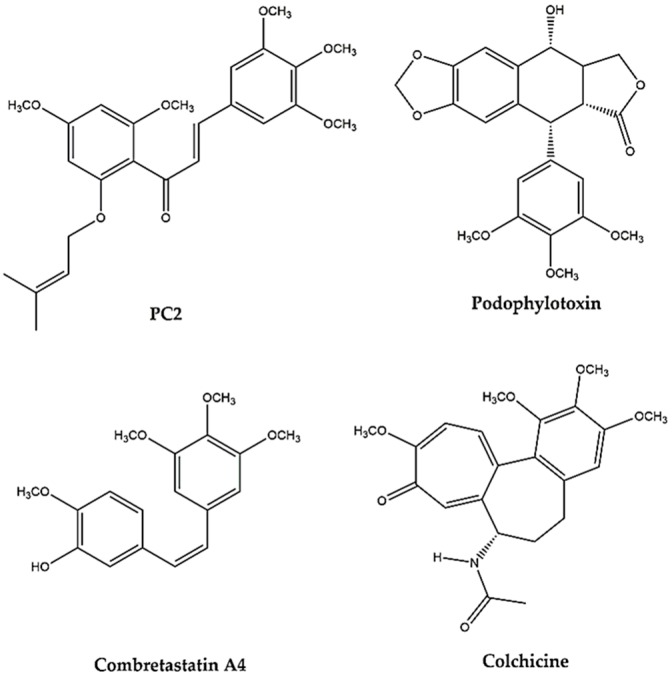
Chemical structures of prenylated chalcone (PC2), podophylotoxin, combretastatin A4 and colchicine.

The molecular recognition phenomenon of MTAs by biological targets has not been explored much. Nevertheless, the presence of a 3,4,5-trimethoxyphenyl moiety in several MTAs, such as podophylotoxin, combretastatin A4 and colchicine (Figure 1), has been much highlighted as playing a crucial rule in the interaction with tubulin [7]. In fact, several compounds possessing 3,4,5-timethoxyphenyl moiety such as trimethoxybenzaldehyde and trimethoxybenzyl alcohol been evaluated for binding with tubulin, and it has been considered that these compounds inhibit the binding of colchicine to tubulin by interaction at the colchicine binding site [8].

Chalcones represent an important class of natural products that are intermediates for the biosynthesis of other classes of flavonoids [9,10]. They are known for their wide range of biological activities, including antitumor activity against a broad spectrum of human cancer cell lines [9,11]. Amongst them, prenylated chalcones, an abundant subclass of flavonoids, had attracted much attention from the scientific community due to their potent bioactivity, namely as promising anticancer agents [12]. Over the last few years, the discovery that chalcones can have antimitotic activity has drawn much attention [13]. Additionally, it has been shown that some compounds containing the chalcone skeleton act as microtubule destabilizing agents, targeting the colchicine binding site [14,15,16,17,18].

We previously reported that the prenylation of chalcone 1 led to the *O*-prenyl derivative PC2 with notably increased cytotoxic activity against tumor cell lines [19]. In addition, in a recent work from our research group using a yeast-based p53-MDM2 interaction assay, combined with assays in HCT116 human tumor cells, the ability of PC2 to disrupt the p53-MDM2 interaction was established [20]. Considering that the chalcone PC2 possess 3,4,5-trimethoxyphenyl moiety described previously as important for the interaction with tubulin, it was hypothesized that this chalcone could also act as antimitotic agent. In this study, we aimed to investigate the antimitotic effect of this chalcone in tumor cells, and explore its ability to enhance chemosensitivity to paclitaxel. We propose chalcone PC2 as a new antimitotic agent with a promising antitumor potential, namely when combined with paclitaxel.

## 2. Results

### 2.1. Treatment with PC2 Arrests MCF-7 and NCI-H460 Cells in Mitosis

**Figure 2:**
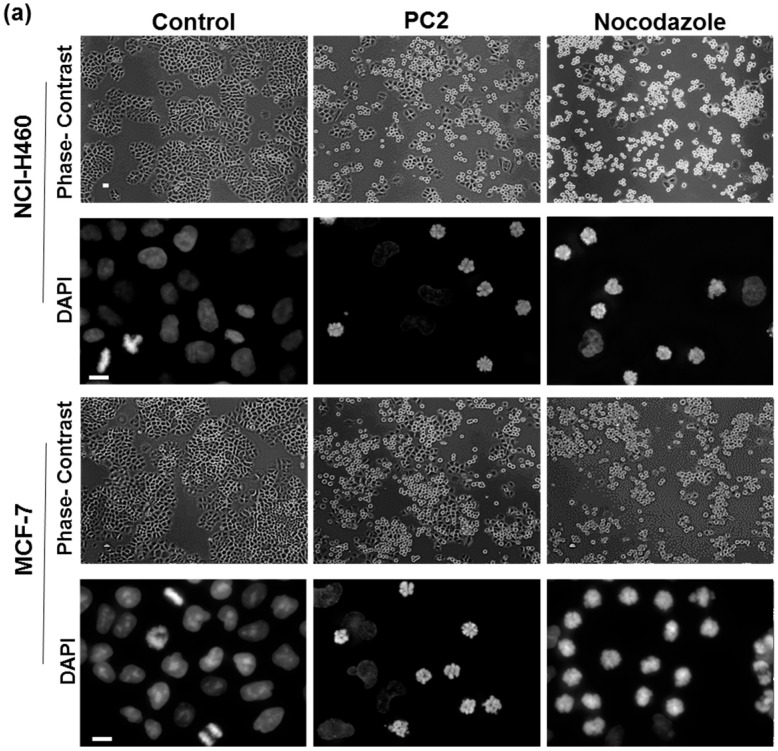

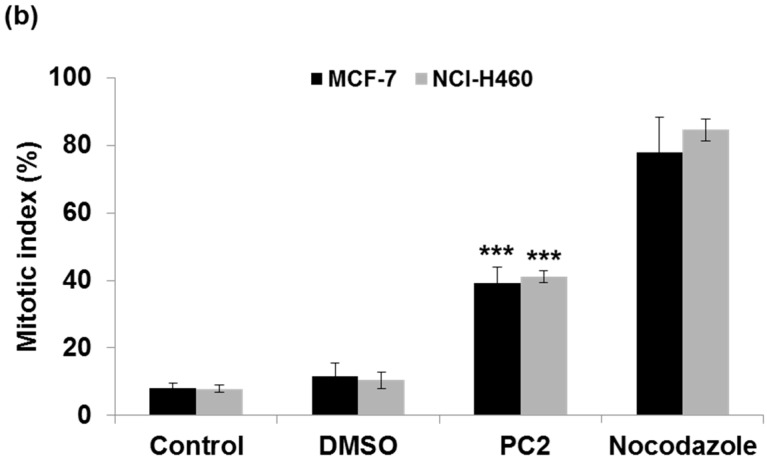
Treatment with PC2 arrests MCF-7 and NCI-H460 cells in mitosis. (**a**) Mitotic cells (rounded) accumulated in cell cultures after 24 h with the compound (PC2), as shown by phase contrast microscopy and confirmed by DAPI staining of DNA. Nocodazole was used as a positive control. Scale bar = 10 μm; (**b**) Mitotic index graph showing accumulation of mitotic cells by 24 h of treatment. (***) represents *p* < 0.001, treated versus DMSO.

The presence in the chalcone PC2 of a 3,4,5-trimethoxyphenyl moiety, known for its ability to interact with tubulin, prompted us to evaluate its effect on mitotic spindle and its antimitotic activity on MCF-7 (breast adenocarcinoma) and NCI-H460 (non-small cell lung cancer) cancer cell lines. We previously determined the GI_50_ value of PC2 as 6.2 ± 0.8 μM and 5.9 ± 0.5 μM for MCF-7 and NCI-H460 cells, respectively [19]. To achieve an efficient effect, it was necessary to use the compound at the concentration of double GI_50_. Light microscopic examination of tumor cells treated for 24 h revealed an accumulation of cells in mitosis (viewed as round and bright cells under phase contrast microscope), which was confirmed by fluorescence microscopy observation of DAPI-stained preparations (Figure 2a). This accumulation was further confirmed by determining the mitotic index by cell-rounding under phase-contrast microscopy: 28.2% of treated MCF-7 and 29.7% of treated NCI-H460 cells were in mitosis contrasting with 8.0% and 7.9% only, in their respective control (Figure 2b). Treatment with nocodazole, a microtubule depolymerizing agent, here used as a positive control of antimitotic activity, increased the mitotic index of MCF-7 and NCI-H460 cells to 78.0% and 84.6%, respectively. We also observed that some levels of cell death occur in PC2-treated culture (see below), which may explain the difference in the mitotic arrest efficiency between PC2 and nocodazole. Therefore, PC2 acts as an antimitotic agent that blocks cell cycle progression in cancer cells.

### 2.2. Treatment with PC2 Induces Mitotic Spindle Collapse

**Figure 3:**
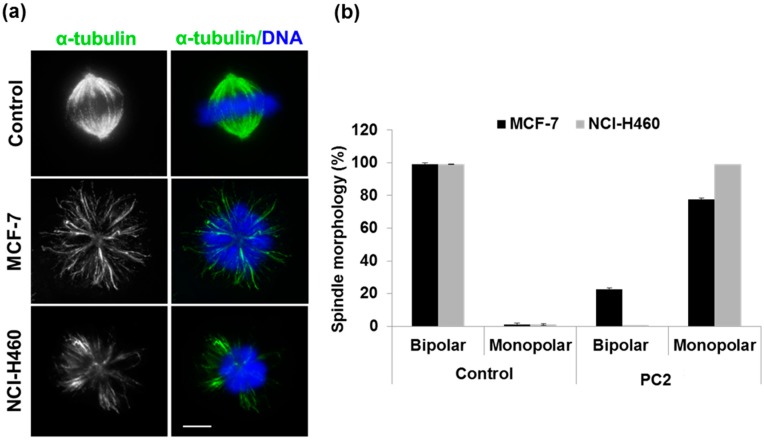

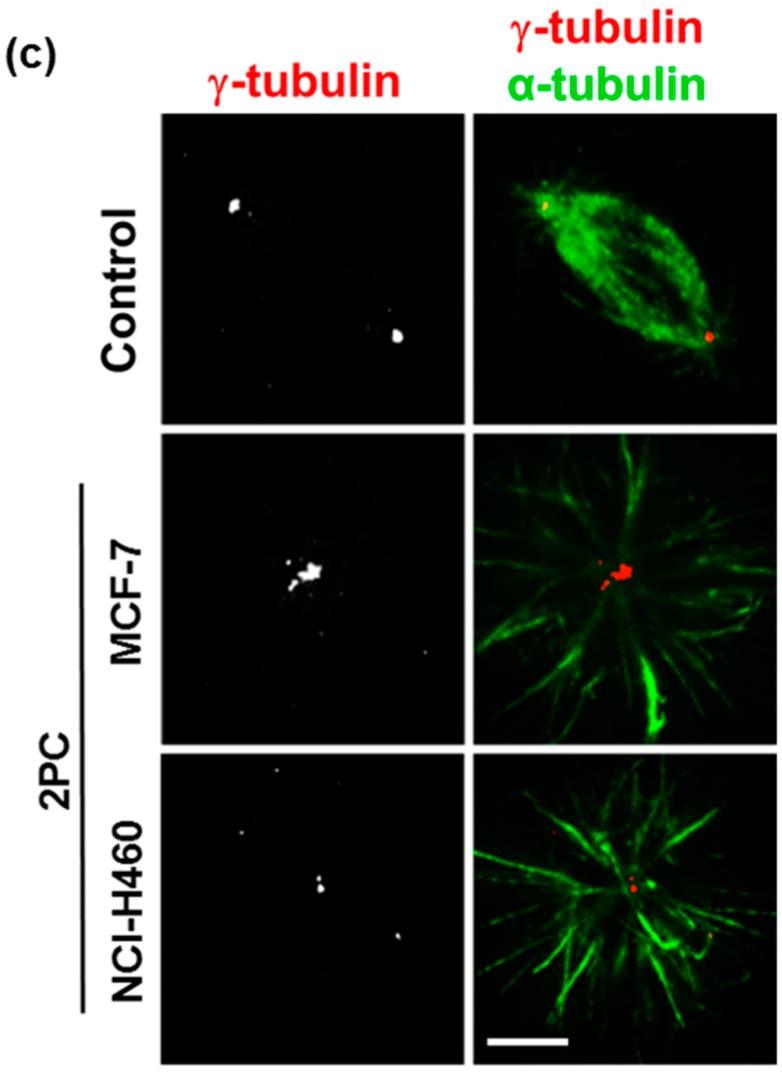
Treatment with PC2 induces mitotic spindle collapse in MCF-7 and NCI-H460 cells. (**a**) Immunofluorescence staining with anti-α-tubulin antibody. Control cells exhibited normal microtubule fibers (green) assembled into a well-organized bipolar mitotic spindle, while treated cells have monopolar spindles. DNA was counterstained with DAPI (blue); (**b**) Graph showing percentage of bipolar and monopolar spindles in control and compound-treated cells; (**c**) Double immunostaining with anti-γ-tubulin (red) and anti-α-tubulin (green) antibodies, showing typical face-to-face poles of a bipolar spindle in control and side-by-side poles of a collapsed spindle in compound-treated cells. Scale bar = 5 μm.

Microtubules are a common target of current antimitotic agents. We investigated whether the PC2-mediated mitotic arrest could be attributed to the disruption of the cytoskeleton. For that, cells were stained for α-tubulin to visualize the mitotic spindle. In contrast to the bipolar and fusiform shape of a typical spindle, found in untreated mitotic cells, monopolar spindles predominated in PC2-treated cells (Figure 3a). Quantification of this phenotype revealed a dramatic increase in the percentage of monopolar spindles, from less than 1% in control cells to more than 77% in PC2-treated cells (Figure 3b). Consistent with monopolar spindle formation in treated cells, γ-tubulin stained the two centrosomes at a single pole, contrasting with their face-to-face configuration in a typical metaphase spindle (Figure 3c). This suggests that the chalcone PC2 affects microtubule structure and compromises the stability of the bipolar spindle, thereby culminating in spindle collapse, consistent with its 3,4,5-trimethoxyphenyl moiety.

### 2.3. Treatment with PC2 Induces Activation of the Spindle Assembly Checkpoint

**Figure 4:**
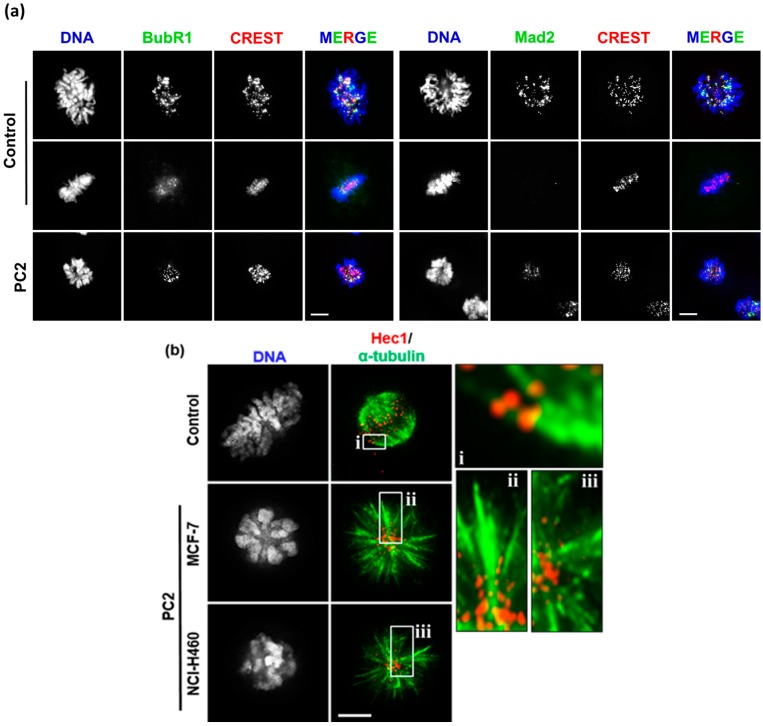
Treatment with PC2 activates the spindle assembly checkpoint in MCF-7 and NCI-H460 cells. (**a**) Immunofluorescence staining with antibodies against Mad2 and BubR1 (green dots), and CREST (red dots) as indicated. In control cells, Mad2 and BubR1 localize on kinetochores at prometaphase (upper panel), and significantly decrease by metaphase (middle panel), consistent with their normal localization pattern. In PC2-treated cells (bottom panel), these proteins are present in all mitotic cells, indicating mitotic checkpoint activation. DNA was counterstained with DAPI (blue). Identical results were obtained for NCI-H460 cells (not shown); (**b**) Double immunostaining showing microtubule fibers (green) attached to kinetochores (Hec1 red dots) in a control metaphase, while microtubules in compound-treated cells do not end on kinetochores indicating that chromosome-to-microtubule attachments are impaired. i, ii and iii insets are representative high magnification images for each condition. Scale bar = 5 μm.

The spindle assembly checkpoint (SAC) is activated in the presence of errors in kinetochore attachments to the mitotic spindle, and acts by arresting cells in mitosis until the defects are successfully repaired, thereby ensuring genomic integrity [21]. We therefore assessed whether PC2-mediated spindle collapse activates the SAC that would provide a molecular explanation as to the mechanism of the antimitotic activity of PC2. SAC activity was assessed in PC2-treated cells by two key SAC markers: Mad2 protein, which localizes at kinetochores solely when these are unattached, therefore serving as an attachment marker; and BubR1 protein, which only leaves kinetochores when they come under tension as the result of opposing forces exerted by microtubules upon bipolar attachment, thus serving as marker for functional attachments [22]. Under fluorescence microscopy, we observed that nearly all mitosis-arrested cells exhibited consistent kinetochore staining for Mad2 and BubR1, which colocalize with the kinetochore marker CREST (Figure 4a). Consistent with this, many kinetochores were not attached to microtubules in PC2-treated cells co-stained for the outer-kinetochore protein Hec1 and for α-tubulin (Figure 4b). This result indicates that monopolar spindles induced upon PC2 treatment create unattached and/or improperly attached kinetochores that lead to SAC activation and blockage of mitotic progression.

### 2.4. Treatment with PC2 Causes a Long Mitotic Delay which Triggers Mitotic Catastrophe Accompanied by Apoptosis

**Figure 5:**
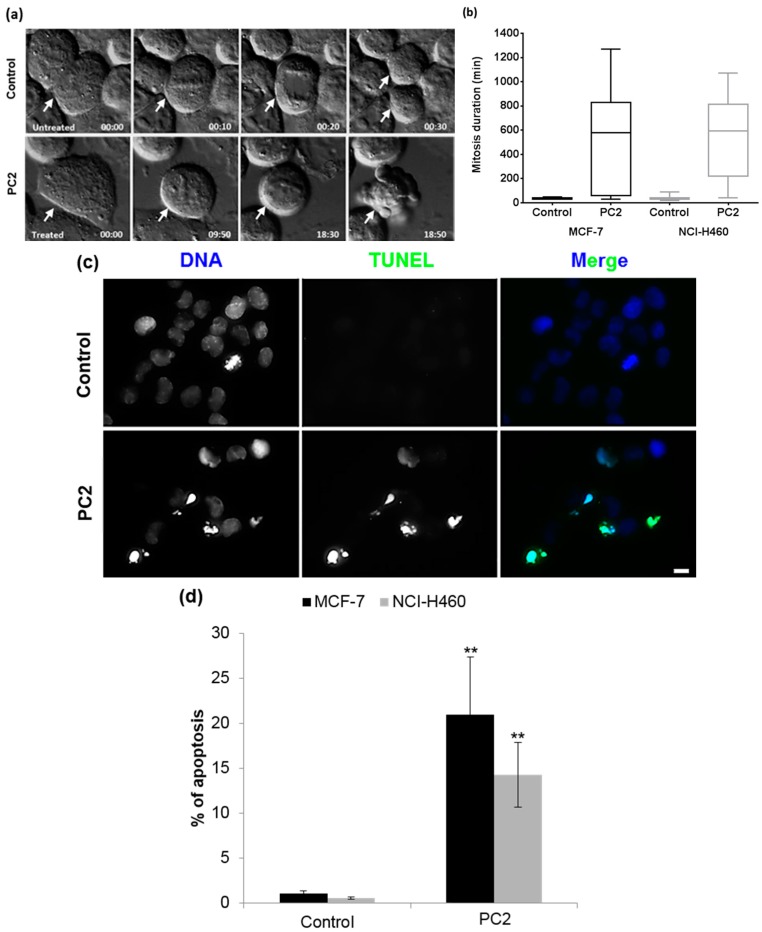
Treatment with PC2 induces mitotic catastrophe and cell death of MCF-7 and NCI-H460 cells. (**a**) Live-cell imaging to determine cell fate in response to compound. Cells were treated with the compound and tracked for up to 48 h. Examples of time-lapse sequences showing a control MCF-7 cell (white arrow, top panel) undergoing normal mitosis giving rise to two daughters, and a treated MCF-7 cell (white arrow, bottom panel) delayed in mitosis and ending in cell blebbing. Numbers indicate times in hours. Similar results were obtained for NCI-H460 cells (not shown). Movies are available as Supplementary Materials (Videos S1 to S4); (**b**) Box plot showing mitosis duration in minutes, in each condition for the two cell lines; (**c**) TUNEL staining showing accumulation of apoptotic cells (green) in MCF-7 cultures incubated with the compound for 48 h. DNA was counterstained with DAPI (blue). Similar results were obtained for NCI-H460 cells (not shown). Scale bar = 5 μm; (**d**) Apoptotic index in control cells and upon 48 h compound treatment, expressed as a percentage of total cells. (**) represents *p* < 0.01.

To further understand the mechanism of PC2-mediated cytotoxicity, we determined the duration of the mitotic arrest and the survival fate of the arrested cells upon PC2 treatment. For that, we performed an analysis at the single cell level, using live-cell imaging with time lapse differential interference contrast (DIC) microscopy, for up to 48 h. Mitosis in untreated MCF-7 (*n* = 53) and NCI-H460 (*n* = 58) cells spent an average of 34.9 ± 5.4 min and 40.0 ± 23.0 min, respectively, from rounding up to separation into two symmetrical daughter cells (Figure 5a,b and videos S1 and S3). PC2-treated cells (*n* = 46) spent on average 13 h in mitosis followed by chromatin condensation, membrane blebbing and cell death, indicative of mitotic catastrophe (Figure 5a,b and Supplementary videos S2 and S4). Cell death by apoptosis was confirmed by the presence of micronuclei, among other abnormalities in nuclear morphology, after DAPI staining (Figure 5c). In addition, Terminal deoxynucleotidyl transferase-mediated nick end labeling (TUNEL) assay revealed that, 30 h after PC2 treatment, asynchronous MCF-7 and NCI-H460 cell cultures accumulated 20.96% and 14.29% of TUNEL-positive cells, respectively, compared to 1.10% and 0.55% in their respective controls (Figure 5c,d). Overall, the results demonstrate that PC2 induces a long mitotic delay culminating in mitotic catastrophe and apoptosis.

### 2.5. Treatment with PC2 Enhances Tumor Cell Sensitivity to Paclitaxel

**Figure 6:**
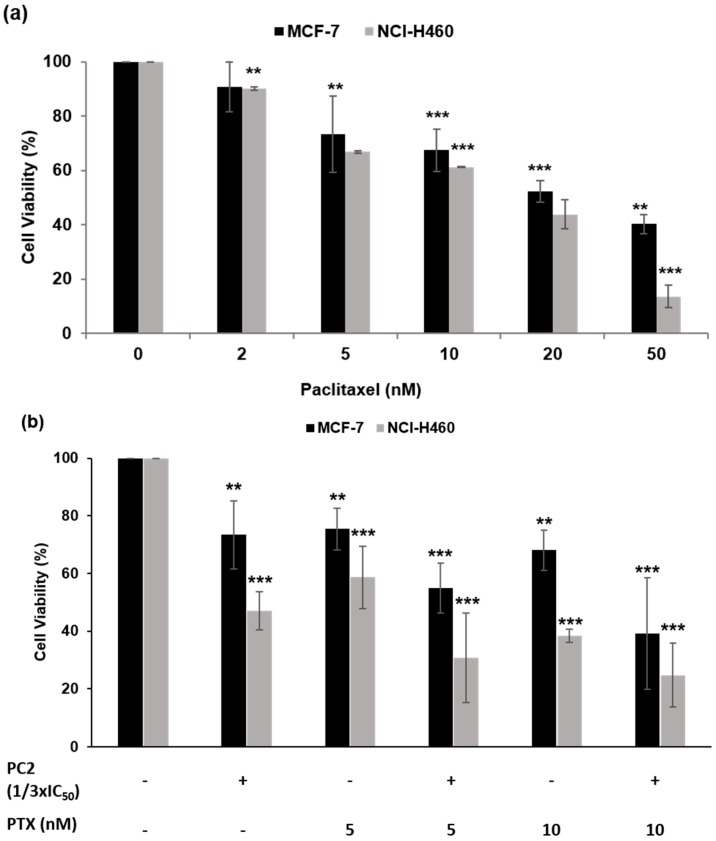

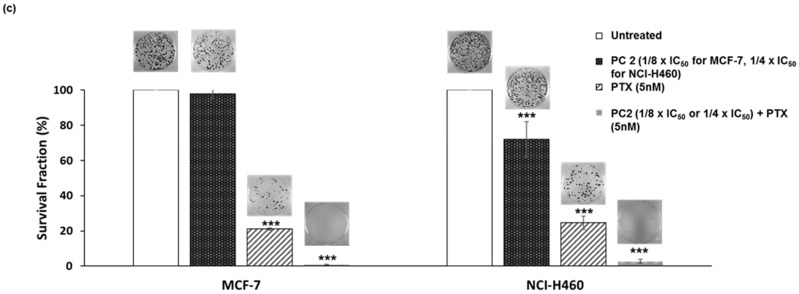
The chalcone PC2 enhances cytotoxicity of cancer cells to paclitaxel (PTX). (**a**) Relative cell viability of MCF-7 and NCI-H460 cells treated with increased concentrations of PTX (0–50 nM) for 48 h; (**b**) Relative cell viability of MCF-7 and NCI-H460 cells treated with PC2 (1/3 × IC_50_) in combination with PTX (5 or 10 nM) for 48 h; (**c**) Colony forming efficiency of MCF-7 and NCI-H460 cells: cells were exposed for 48 h to PC2, PTX, or the combination of both, washed, and allowed to grow in fresh medium for 10 days before crystal violet staining. Representative figures are shown and viable colonies, of at least 50 cells each, (top) were counted and the results expressed as % survival fraction relative to control (bottom). Results are expressed as mean ± standard deviation (SD), from at least three independent experiments. (***) represents *p* < 0.001; (**) represents *p* < 0.01, PC2 and/or PTX versus control.

We asked if PC2 might enhance chemosensitivity and efficacy of tumor cells to paclitaxel (PTX). The rational of this approach is that the two drugs should target different aspects of the mitotic spindle and thus, mutually increase their efficacy in terms of antimitotic activity and cytotoxicity. According to the results of cell viability, at 48 h post-treatment, using the MTT assay, the calculated IC_50_ of PC2 was 12.48 ± 0.29 and 3.20 ± 0.63 μM for MCF-7 and NCI-H460, respectively, and that of PTX was 10.38 ± 0.34 and 10.20 ± 0.44 nM for MCF-7 and NCI-H460, respectively (Figure 6a). We found that after treatment of MCF-7 cells with 1/3 × IC_50_ concentration of PC2 + 10 nM PTX, over 60% decrease of cell viability was detected, while nearly 30% or 40% decrease of cell viability only was observed after individual treatment with PC2 or PTX, respectively (Figure 6b). For NCI-H460 cells, a PC2 (1/3 × IC_50_) + 5 nM PTX combination resulted in more than 80% decrease of cell viability (Figure 6b). The efficacy of the combination was much more obvious when we assessed its ability to inhibit cancer cell proliferation in a long-term (10 days) clonogenic assay. For instance, combination of doses as low as 1/8 × IC_50_ PC2 + 5 nM PTX were able to almost completely abolish colony formation in MCF-7 tumor cell lines (Figure 6c). These observations indicate that the chalcone PC2 increases the cytotoxic effect of paclitaxel and render tumor cells more sensitive to low doses of both antimitotic agents.

## 3. Discussion

The present study sheds light onto the mechanism underlying the previously reported cytotoxic effect of the prenylated chalcone PC2, in MCF-7 (breast adenocarcinoma) and NCI-H460 (non-small cell lung cancer) cancer cells. The data showed that PC2 exerts its antiproliferative activity by inducing the collapse of the mitotic spindle. This activates the spindle assembly checkpoint thus leading to prolonged mitotic arrest and subsequent cell death by apoptosis. In addition, the compound enhanced sensitivity of the referred cancer cell lines to paclitaxel.

Treatment of MCF-7 and NCI-H460 cells with PC2 disrupted the organization of the mitotic spindle culminating in its collapse. One should expect PC2 to induce microtubule disassembly, like other chalcones belonging to the class of colchicine-binding agents. We believe that the compound might not affect microtubule assembly as seriously as colchicine does, probably because its chemical structure still needs to be improved. This mild effect may explain why microtubules did not depolymerize as expected but, instead, their dynamic is affected in a way that compromises spindle bipolarity. It is well known that colchicine suppresses microtubule dynamics at lower doses and induces their depolymerization at higher doses [23]. Increasing PC2 concentrations did not lead to microtubule depolymerization, thereby stressing the need to improve its chemical structure.

The monopolar-like configuration of the mitotic spindle induced by PC2 treatment created unattached or improperly attached chromosomes which, in turn, activated the SAC as evidenced by the kinetochore accumulation of the SAC proteins Mad2 and BubR1. Consequently, PC2-treated cells accumulated in state of mitosis. Thus, PC2 acts as an antimitotic agent against MCF-7 and NCI-H460 cancer cells by disrupting mitotic spindle organization.

Cells are thought to gradually accumulate pro-apoptotic signals culminating in their death after a prolonged arrest in mitosis following exposure to antimitotic agents [24,25]. Our live-cell imaging analysis indicated that the great majority of PC2-treated cells experienced a long-term arrest in mitosis followed irreversibly by apoptotic cell death, as evidenced by membrane blebbing and also by DAPI and TUNEL staining. Cell death that occurs following a degree of mitotic arrest due to perturbation of the mitotic spindle defines the cell death process known as mitotic catastrophe [26,27,28]. Although the mechanisms that drive mitotic catastrophe remain poorly understood, functionally it is viewed as an oncosuppressive mechanism prior to cell death by apoptosis, necrosis or senescence [28]. Induction of mitotic catastrophe precipitates the elimination of mitosis-incompetent cell, thereby constituting a valuable therapeutic strategy against cancer. We then propose that PC2 induces mitotic catastrophe in MCF-7 and NCI-H460 cancer cells, revealing a potential oncosuppressive activity. A common alternative cellular response to microtubule poisoning is mitotic slippage, when cells exit mitosis and become tetraploid after a prolonged mitotic arrest, and is considered as a cause of drug resistance [24,29].

It was proposed that mitosis-arrested cells, upon exposure to MTAs, start accumulating a death signal and, in parallel, degrading cyclin B, until a threshold is reached that determines if cells will die or undergo slippage [29]. The event that reaches first its threshold will determine the fate of the mitosis-arrested cells: if cyclin B is degraded below its threshold level first, then cells will not sustain the mitotic arrest and undergo slippage; otherwise, if the threshold of cell death signal is reached first, then cells will die. In our single-cell imaging analysis, slippage of cells arrested in mitosis by PC2 was rather occasional in both cancer cell lines, apoptosis being the predominant cell fate. This result is in good agreement with the role of PC2 in p53 induction. Indeed, using yeast-based assays and human in vitro systems, we recently reported that PC2 disrupts the p53-MDM2 interaction, leading to upregulation of the pro-apoptotic Bax, and increased poly(ADP-ribose) polymerase (PARP) cleavage [20]. We therefore suggest that PC2 affects microtubule function, probably through its 3,4,5-trimethoxyphenyl moiety, resulting in spindle collapse, which induces prolonged mitotic arrest, and, subsequently, promotes p53 induction leading to apoptosis. Indeed, prolonged arrest in mitosis has been reported to trigger partial activation of apoptosis, resulting in DNA damage and p53 induction [29,30,31]. This would explain why apoptosis is the predominant fate of cells arrested in mitosis due to PC2 treatment: the threshold of the death signal would be reached well before that of cyclin B degradation.

Our study defines an important role of the chalcone PC2 in enhancing chemosensitization of paclitaxel in tumor cells: the 3,4,5-trimethoxyphenyl moiety of PC2 predicts a microtubule binding site different from that of paclitaxel, thereby enhancing the effect of paclitaxel on microtubule assembly. Moreover, the fact that PC2 promotes p53 induction would enhance the responsiveness of tumor cells to paclitaxel by promoting apoptosis at the expense of slippage. Therefore, treatment strategies that combine paclitaxel with PC2 may have therapeutic benefits attributable to paclitaxel chemosensitization through p53-mediated apoptosis. It is noteworthy that the PTX concentrations used are within the lower range (~5 nM) of what is considered to be clinically relevant doses in cell culture (<100 nM), strengthening further the potential effectiveness of the compound in a combinatorial treatment [32,33]. In our future studies, we will evaluate the in vivo impact of this combination on the effectiveness of paclitaxel in a mouse model of human cancer.

## 4. Materials and Methods

### 4.1. Chemicals

Prenylated Chalcone PC2 synthesized as previously described was dissolved in Dimethyl Sulfoxide (DMSO; Sigma-Aldrich, St. Louis, MO, USA) to a stock concentration of 60 mM and stored at −20 °C [19]. Nocodazole and paclitaxel (Sigma-Aldrich) were dissolved in DMSO as a 0.5 mM stock. The desired concentrations were freshly prepared prior to each experiment.

### 4.2. Cell Culture

MCF-7 breast adenocarcinoma and NCI-H460 non-small cell lung cancer human cell lines (European Collection of Cell Culture, Salisbury, Wiltshire, UK) were grown in RPMI-1640 culture medium (Lonza, Basel, Switzerland), supplemented with 5% fetal bovine serum (FBS), and cells were maintained at 37 °C in a 5% CO_2_ humidified atmosphere.

### 4.3. Viability Assay

Cells were plated in 96-well plates (0.05 × 10^6^ cells/well) in complete culture medium and incubated at 37 °C for 24 h. Cells were then incubated for 48 h with the test compound, paclitaxel, or a combination of both (see the Results section for concentrations used) at 37 °C and 5% CO_2_. Then, cells were placed in fresh serum-free medium, and MTT (3-(4,5-dimethylthiazol-2-yl)-2,5-diphenyltetrazolium bromide), previously dissolved in phosphate-buffered saline (PBS), was added to each well (0.5 mg/mL) and incubated for 4 h at 37 °C and 5% CO_2_. Formazan crystals were then solubilized by adding 100 μL solubilization solution (89% Isopropanol, 10% of Triton-100, 0.37% HCl) for 2 h. Absorbance was measured at 570 nm in a microplate reader (Biotek Synergy 2, Winooski, VT, USA), and retrieved through the Gene5 software (version 1.07.5, Biotek). Viability was calculated relative to untreated cells.

### 4.4. Clonogenic Assay

A total of 750 cells were seeded in six-well plates, allowed to attach for 24 h, and treated with PC2 for 48 h with indicated concentrations. Cells were washed twice with PBS and then incubated in a drug-free RPMI medium for 10 days. Grown colonies were fixed for 5 min with 3.7% (*w*/*v*) paraformaldehyde in PBS and stained for 20 min with 0.05% (*w*/*v*) Violet Crystal (Merck Millipore, Billerica, MA, USA) in distilled water. For each condition, the number of colonies was counted on duplicate dishes from three independent experiments. The plating efficiency (PE) was calculated as the percentage of the number of colonies formed over the number of cells seeded. The survival fraction was determined as the number of colonies over the number of cells seeded × PE.

### 4.5. Determination of Mitotic Index

MCF-7 or NCI-H460 cells (2 × 10^5^) were grown in six-well dishes and treated for 24 h with 12 μM PC2. Treatment with 1 μM nocodazole served as a positive control. Controls included untreated cells and cells treated with the highest concentration of DMSO used to dissolve the compounds. Mitotic index, percentage of mitotic cells over total cell population, was determined by cell-rounding under phase-contrast microscopy [22]. For each condition, a total of 2000 mitotic and interphasic cells were counted from more than ten random microscope fields.

### 4.6. Immunofluorescence

MCF-7 and NCI-H460 cells were seeded on 22 mm poly-l-lysine-coated coverslips, into a 6-well plate, and incubated with 12 μM PC2. Twenty-four hours later, cells were fixed in fresh 2% (*w*/*v*) paraformaldehyde (Sigma-Aldrich) in PBS for 12 min, rinsed three times in PBS for 5 min each, and permeabilized with 0.5% Triton X-100 (Sigma-Aldrich) in PBS for 7 min. Alternatively, to stain microtubules, cells were fixed in cold methanol at −20 °C for 10 min, and rehydrated twice in PBS. Then, cells were blocked with 10% FBS in PBST (0.05% Tween-20 in PBS for 30 min at room temperature, followed by 1 h incubation at room temperature with the primary antibodies, diluted in PBST containing 5% FBS. The primary antibodies used were: mouse anti-BubR1 (1:400, Chemicon International, Temecula, CA, USA); mouse anti-Mad2L1 (1:200, Sigma-Aldrich); mouse anti-Hec1 (1:600, Abcam, Cambridge, UK); rabbit anti-α-Tubulin (1:100 and 1:300 in paraformaldehyde or methanol, respectively, Abcam); mouse anti-α-Tubulin (1:2500, Sigma-Aldrich) and mouse anti-γ-Tubulin (1:1500, Sigma-Aldrich). After washing in PBST, cells were probed with Alexa Fluor 488 (Molecular Probes, Eugene, OR, USA) and/or 568 conjugated secondary antibodies for 1 h. All secondary antibodies were diluted at 1:1500 in PBST with 5% FBS. Cover glasses were mounted in Vectashield mounting medium (Vector, H-1000, Burlingame, CA, USA) containing 2 μg/mL DAPI for DNA staining. Preparations were observed in an Axio Observer Z1 fluorescence microscope (Carl Zeiss, Oberkochen, Germany).

### 4.7. TUNEL Assay

To evaluate the ability of PC2 to induce apoptosis, and TUNEL assay was performed using DeadEnd Fluorometric TUNEL System (Promega, Madison, WI, USA). MCF-7 and NCI-H460 cells were treated with PC2 (2 × GI_50_) for 30 h, processed as previously described, and then subjected to TUNEL assay according to the manufacturer’s instructions [34]. DNA was stained with 2 μg/mL DAPI in Vectashield mounting medium (Vector, H-1000, Burlingame, CA, USA). The level of apoptotic cells was determined by scoring TUNEL-positive cells in a total of 400 cells under fluorescence microscope, from at least five separate randomly selected microscopic fields, for each experimental condition.

### 4.8. Time-Lapse Video Microscopy

Live-cell imaging experiments were performed essentially as previously described [34]. Briefly, MCF-7 or NCI-H460 cells were seeded onto LabTek II chambered cover glass (Nunc, Penfield, NY, USA) containing 1 mL of RPMI supplemented with 5% FBS, and incubated overnight at 37 °C under 5% CO_2_. Then, the medium was replaced with 1 mL of RPMI without phenol red supplemented with 5% FBS, in the presence of 12 μM of the compound. Images were captured at 10 min intervals for 48 h under differential interference contrast (DIC) optics, with a 63x objective on an Axio Observer Z.1 SD inverted microscope (Carl Zeiss), equipped with an incubation chamber with the temperature set to 37 °C and an atmosphere of 5% CO_2_. Movies were generated from the time-lapse images using ImageJ software (version 1.44, Rasband, W.S., ImageJ, U. S. National Institutes of Health, Bethesda, MD, USA). The number of cells that were arrested at mitosis, apoptotic, or bypassed cytokinesis was scored.

### 4.9. Image Acquisition and Processing

Phase-contrast microscopy images were acquired with a 10x objective, on a Nikon TE 2000-U microscope (Amsterdam, The Netherlands), using a DXM1200F digital camera (Amsterdam, The Netherlands) and with Nikon ACT-1 software (version 2.62, Melville, NY, USA). Fluorescence images were acquired with Plan Apochromatic 63x/NA 1.4 objective on an Axio Observer Z.1 SD microscope, coupled to an AxioCam MR3 digital camera (Carl Zeiss). Z-stacks were acquired with 0.4 μm intervals and images were processed using ImageJ software, after deconvolution with AxioVision Release SPC software (version 4.8.2, Carl Zeiss).

### 4.10. Statistical Analysis

Data are presented as the means ± SD of at least three independent experiments (at least three replicates each). Differences between the experimental groups and the control groups were assessed using an unpaired student’s *t*-test. Values of differences with *p* < 0.05 were considered significant.

## 5. Conclusions

In summary, our work adds valuable data on the characterization of the mechanism of action of the prenylated chalcone PC2, as well as to its potential as antitumor agent either individually or in combination with paclitaxel. We demonstrate that the prenylated chalcone PC2 induces a prolonged and SAC-dependent mitotic arrest by interfering with mitotic spindle assembly, which ultimately leads to cell death. We thus propose PC2 as a new antimitotic agent with a great potential as an oncosuppressive drug. The combination of this antimitotic effect with the previously described role in disrupting p53-MDM2 interaction makes this chalcone a promising potential multitarget antitumor agent. Additionally, our findings highlight the importance of PC2 as a promising drug candidate for improving the efficacy of paclitaxel-based chemotherapy. Further studies aiming to explore this multitarget antitumor potential of PC2 are under investigation.

## Supplementary Materials

Supplementary materials can be accessed at: http://www.mdpi.com/1420-3049/21/8/982/s1.
